# Isolation of a Moderately Acidophilic *Nitrobacter* from a Nitrifying Community Supplied with Urea

**DOI:** 10.1264/jsme2.ME24027

**Published:** 2024-09-13

**Authors:** Yuta Endo, Hirotsugu Fujitani, Ayano Kaneko, Takuya Ninomiya, Chiharu Umezawa, Megumi Kuroiwa, Yuichi Suwa

**Affiliations:** 1 Department of Biological Sciences, Chuo University, Tokyo, Japan

**Keywords:** acidic pH, isolation, nitrite-oxidizing bacteria, soil, urea

## Abstract

Nitrite-oxidizing bacteria (NOB), which perform the second step of aerobic nitrification, play an important role in soil. In the present study, we report a novel isolate from agricultural soil affiliated with the genus *Nitrobacter* and its physiological characteristics. We sampled the surface soil of a vegetable field and obtained mixed culture A31 using the most probable number (MPN) method with inorganic medium containing 0.75‍ ‍mM urea (pH 5.5). The dilution–extinction procedure on culture A31 led to the isolation of a strain that was designated as *Nitrobacter* sp. A67. The *nxrB1* gene sequence of *Nitrobacter* sp. A67 (302 bp) was classified into Cluster 5, and the highest sequence identity was 96.10% with *Nitrobacter* sp. BS5/19. The NO_2_^–^ oxidation activity of *Nitrobacter* sp. A67 was investigated at various pH. The optimum pH for NO_2_^–^ oxidation was 5.8–6.4. This result indicates that *Nitrobacter* sp. A67 is a moderately acidophilic nitrite-oxidizing bacterium.

Nitrification, the biological oxidation of ammonia (NH_3_) to nitrate (NO_3_^–^) in the global nitrogen cycle, is regarded as a major pathway for supplying NO_3_^–^, a rate-limiting nutrient source for primary production in soil and aquatic ecosystems. The nitrification process often results in environmental issues, such as the eutrophication of aquatic systems due to NO_3_^–^ outflow, greenhouse gas (mainly N_2_O) emission, and nitrogen loss from agricultural soils (Robertson *et al.*, 2013; Norton and Ouyang, 2019; Prosser *et al.*, 2020). Nitrification typically consists of two steps: the oxidation of NH_3_ to nitrite (NO_2_^–^) by ammonia-oxidizing bacteria (AOB) and ammonia-oxidizing archaea (AOA), collectively designated as ammonia-oxidizing microorganisms (AOM), and the consecutive oxidation of NO_2_^–^ to NO_3_^–^ by nitrite-oxidizing bacteria (NOB). Therefore, NO_2_^–^ rarely accumulates in the environment. *Nitrospira* that mediate the complete ammonia oxidation (comammox) of NH_3_ to NO_3_^–^ have recently been discovered (Daims *et al.*, 2015; van Kessel *et al.*, 2015).

In the soil NOB community, the importance of *Nitrobacter* and *Nitrospira* as two NOB genera that are more abundant and sensitive to agricultural management has been recognized (Han *et al.*, 2018). In paddy field soil, the abundance of *Nitrobacter*-like NOB increased in response to nitrogen levels, whereas *Nitrospira*-like NOB predominated in nutrient-limited surface soil (Ke *et al.*, 2013). Potential nitrite oxidation (PNO) in tilled/no-tillage agricultural soils with high nitrogen availability positively correlated with the abundance of *Nitrobacter*, but negatively correlated with that of *Nitrospira*-like NOB (Attard *et al.*, 2010). Long-term fertilization in acidic forest soil increased the abundance of *Nitrobacter*-like NOB, but did not markedly affect that of *Nitrospira*-like NOB (Wertz *et al.*, 2012). In acidic agricultural soils (pH 4.92) fertilized with urea, the abundance of *Nitrospira*-like NOB exceeded that of *Nitrobacter*-like NOB (Wang *et al.*, 2014a). As described above, N availability markedly affected the abundance of *Nitrobacter* and *Nitrospira* and pH also had an impact on NOB.

Ni *et al.* (2023) recently reviewed nitrification in acidic and alkaline environments. Soil pH has been regarded as an important factor affecting the activity, population, and diversity of nitrifying microorganisms in the environment (Li *et al.*, 2019). The abundance of AOM in acidic environments and the physiology of AOM isolates from acidic agricultural soils have been extensively exami­ned (*e.g.* Nicol *et al.*, 2008; Lehtovirta-Morley *et al.*, 2011; Lehtovirta-Morley *et al.*, 2014; Hayatsu *et al.*, 2017). Previous studies reported the abundance of NOB at the genus level in acidic soil (*e.g.* Wertz *et al.*, 2012; Wang *et al.*, 2014a) and comammox *Nitrospira* was enriched in acidic soil from a tea field (Takahashi *et al.*, 2020). However, to the best of our knowledge, only three strains of the genus *Nitrobacter*, *Nitrobacter* sp. Io acid, *Nitrobacter winogradskyi*, and *Nitrobacter* sp. strain NHB1, were shown to be capable of oxidizing nitrite at acidic pH in cultures (Bock and Heinrich, 1969; Hankinson and Schmidt, 1988; De Boer *et al.*, 1991). Therefore, the isolation of novel NOB with ecologically relevant characteristics remains an important challenge.

The majority of previous NOB isolates were obtained from cultures containing NO_2_^–^ as the sole nitrogen and energy source for NOB (*e.g.*, Watson *et al.*, 1986; Hankinson and Schmidt, 1988; Sorokin *et al.*, 1998; Spieck *et al.*, 2014). In some cases, NH_3_ was used as the sole nitrogen and energy source in co-cultures with AOM (Watson and Waterbury, 1971; Sorokin *et al.*, 2014; Fujitani *et al.*, 2020). As shown in Fig. S1, in acidic pH, free ammonia, NH_3_ (FA) decreased (NH_4_^+^+OH^–^⇌NH_3_+H_2_O), whereas free nitrous acid, HNO_2_ (FNA) increased (H^+^+NO_2_^–^⇌HNO_2_) due to chemical equilibria (Anthonisen *et al.*, 1976). HNO_2_ and a high concentration of NH_3_ were previously reported to inhibit NOB and AOB (Kim *et al.*, 2006; Wang *et al.*, 2014b; Zhang *et al.*, 2018; Zheng *et al.*, 2021). When urea is supplied to a nitrification community, it is decomposed into NH_3_ either/both by heterotrophic bacteria and/or urease-positive nitrifiers, and NH_3_ may then be intracellularly oxidized to NO_2_^–^ by AOM. The NO_2_^–^ produced may be consecutively oxidized to NO_3_^–^ by NOB. Therefore, the potential inhibitory effects of free NH_3_ and HNO_2_ on NOB may be suppressed, and acidic NOB may then be more easily isolated.

We herein report the isolation of a novel *Nitrobacter* strain in agricultural soil that accommodates a moderately acidic pH. The aims of the present study were as follows: i) to obtain a nitrifying community in which complete nitrification progresses to NO_3_^–^ with urea, and ii) to isolate acidophilic *Nitrobacter* from the nitrifying community by dilution-extinction with NO_2_^–^ as the sole nitrogen and energy source.

## Materials and Methods

### Soil sample

Soil was sampled on October 2, 2017 from an open field for cultivating vegetables, such as onions, carrots, and sweet corn, which was supplied for more than 20 years with organic compost made mainly from cow dung and residues at Tsukuba, Ibaraki. Five kilograms of soil was obtained at a depth of 10‍ ‍cm from the field surface, placed in a cooler box with ice, and transferred to the laboratory. Soil subsamples were placed in plastic bags or glass containers with screw caps and stored at 4°C until used. Soil pH was measured using the H-SERIES H160 pH meter (HACH). Measurements were performed with a water/soil (v/w) ratio of 2.5. The pH of soil samples was 7.2 (Table S1). Other soil chemical properties are shown in Supplementary materials (Table S1)

### Media

In the present study, we used a medium modified from the previously described basal medium C (Suwa *et al.*, 1994) by adding 220‍ ‍mg L^–1^ NaHCO_3_ as a carbon source due to the reduced CO_2_ supply under acidic conditions and by lowering the amount of the trace element mixture in ATCC medium #1573 from 1.0 to 0.6‍ ‍mL L^–1^. ULAC medium (pH 5.5), containing 0.75‍ ‍mmol L^–1^ urea in modified basal medium C, and UL medium (pH 7.6) were used for most probable number (MPN) enumeration. ULAC medium was used for the subculture of culture A31. NUAC medium (pH 5.5), containing 100‍ ‍μmol L^–1^ NaNO_2_ in modified basal medium C as the sole source of nitrogen and energy for NOB, was used to isolate NOB (pH 5.5). Detailed information is provided in Supplementary materials.

In physiological studies on *Nitrobacter* sp. A67, the isolate designated in the present study, modified basal medium C containing 0.75‍ ‍mmol L^–1^ NaNO_2_ was dispensed into eight bottles and citric acid and Na_2_HPO_4_ were added as the buffer of this medium. pH was adjusted to 3.7, 4.2, 4.7, 5.3, 5.8, 6.4, 6.8, and 7.3 based on the concentration ratio of citric acid and Na_2_HPO_4_. Media were sterilized by filtration using a membrane filter with a pore size of 0.2‍ ‍μm. To select the optimum temperature, sterile NUAC medium (pH 5.5) containing 0.75‍ ‍mmol L^–1^ NaNO_2_ was used.

### MPN enumeration

One gram of the soil sample was fully suspended in 9‍ ‍mL of sterile ULAC medium. The soil suspension (3‍ ‍mL, 10-fold diluted suspension) was transferred to 27‍ ‍mL sterile ULAC medium (10^2^-fold diluted suspension) and was thoroughly resuspended. This procedure was repeated to prepare a 10^3^- to 10^6^-fold diluted suspension. Subsequently, 0.5-mL aliquots of each dilution (10^2^- to 10^6^-fold) were transferred to small test tubes containing 4.5‍ ‍mL of sterile ULAC medium in five replicates for MPN enumeration. The same method was performed with UL medium for MPN enumeration. After an incubation at 25°C for 42 days, NO_2_^–^ and NO_3_^–^ concentrations were measured using the colorimetric method (Miranda *et al.*, 2001). Cultures were regarded as ‘AOM-positive’ and ‘AOM+NOB-positive’ when NO_2_^–^ and NO_3_^–^ concentrations, respectively, were higher than 10‍ ‍μM.

### Isolation of NOB

**Fig. 1.**
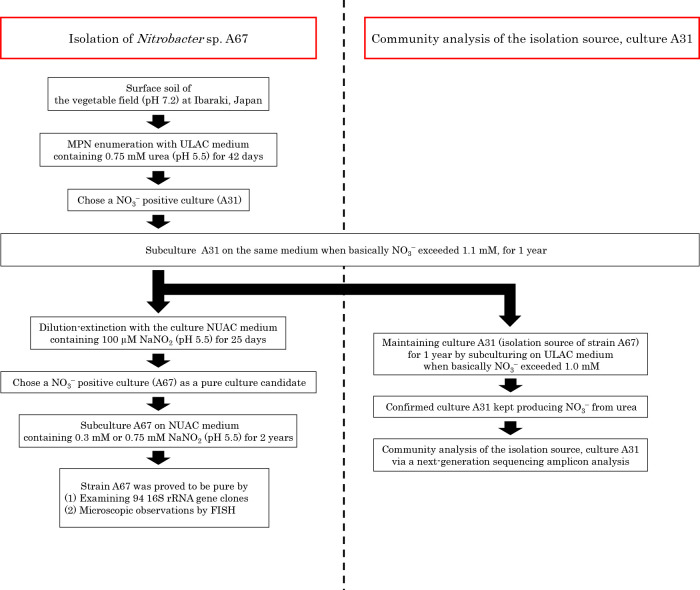
Flow chart of this study.

The NOB pure culture candidate (A67) was isolated from mixed culture A31 via a dilution-extinction procedure employing NUAC medium. An aliquot of fresh medium from culture A31 was diluted 10^8^-fold with sterile NUAC medium containing 100‍ ‍μmol L^–1^ NaNO_2_ as the sole source of nitrogen and energy for NOB, and 0.3-mL aliquots were transferred to 400 small test tubes containing 2.7‍ ‍mL of sterile NUAC medium, which made a 10^9^-fold dilution of the culture. After an incubation at 25°C for 25 days, NO_3_^–^ concentrations were measured using the colorimetric method, and NO_3_^–^ positivity was assessed by the naked eye. The flow of procedures to isolate NOB is shown in Fig. 1.

### Physiological characterization of the isolate

To select the optimum pH for NO_2_^–^ oxidation, *Nitrobacter* sp. A67 was incubated at various pH (3.7, 4.2, 4.7, 5.3, 5.8, 6.4, 6.8, 7.3) at 25°C for 17 days. *Nitrobacter* sp. A67 was precultured without stirring in 135‍ ‍mL of modified NUAC medium (pH 5.5) containing 0.75‍ ‍mmol L^–1^ NaNO_2_ in a 300-mL Erlenmeyer flask at 25°C at 10% (v/v). Sterile NaNO_2_ solution was added to the fed-batch. When the culture consumed 1.5‍ ‍mM NO_2_^–^, 5‍ ‍mL of the preculture were transferred to freshly prepared medium consisting of 45‍ ‍mL of sterile modified basal medium C with various pH values and 0.75‍ ‍mM NaNO_2_ in 100-mL Erlenmeyer flasks. NO_2_^–^ and NO_3_^–^ concentrations and pH were assessed throughout the incubation period. Experiments were performed in duplicate. To select the optimum temperature for NO_2_^–^ oxidation, *Nitrobacter* sp. A67 was incubated at various temperatures (10, 15, 20, 25, 30, 37, and 42°C) for 16 days using the same method as the pH test. Experiments were performed in triplicate.

### Chemical ana­lysis

Nitrite and nitrate concentrations were measured by a colorimetric method using the Enspire Multimode Plate Reader (Perkin Elmer). Nitrite concentrations were quantified by coloring with Griess reagent (Miranda *et al.*, 2001) and measuring absorbance at‍ ‍538‍ ‍nm. Nitrate concentrations were assessed as nitrite after reducing nitrate with vanadium (III) solution (Miranda *et al.*, 2001).

### Purification check

Strain A67 was proven to be pure by (1) an examination of 94 16S rRNA gene clones using the primer set 27f/1492r (Table S2), and (2) microscopic observations by fluorescence *in situ* hybridization (FISH) with a genus-specific probe (Table S3) and (3) 4 media commonly used to cultivate heterotrophs. Detailed information is provided in Supplementary material.

### PCR and sequencing ana­lysis

A microbial community ana­lysis of culture A31 (isolation source of strain A67), which was maintained for 1 year by subculturing on ULAC medium (pH 5.5), was performed using a next-generation sequencing amplicon ana­lysis targeting the 16S rRNA gene (TechnoSuruga Laboratory). Briefly, DNA was extracted from culture A31 using ISOIL for the Beads Beating kit (Nippon Gene). Extracted DNA was amplified by PCR with a primer set targeting the V3–V4 region of the 16S rRNA gene. The amplicon was sequenced using MiSeq (Illumina) and MiSeq Reagent Kit v3 (Illumina). The sequence was analyzed using the Ribosomal Database Project (RDP) and DB-BA 13.0 (TechnoSuruga Laboratory). Sequences with homology of 97% or higher were compiled as operational taxonomic units (OTUs).

In the PCR amplification of the bacterial 16S rRNA gene sequences of strain A67, the primer set 27f/1492r (Table S1) was used with TaKaRa Ex Taq (TaKaRa Bio) according to the following thermal protocol: at 94°C for 30 s; 35 cycles at 98°C for 10‍ ‍s, 56°C for 30‍ ‍s, and 72°C for 90 s; and at 72°C for 5‍ ‍min. In the PCR amplification of the *nxrB1* gene sequences of strain A67, the primer set nxrB-1F/1R (Table S1) was used with the following thermal protocol: at 95°C for 10‍ ‍min; 35 cycles at 98°C for 10‍ ‍s, 55°C for 90‍ ‍s, and 72°C for 1‍ ‍min; and at 72°C for 12‍ ‍min. The nearly full lengths of bacterial 16S rRNA gene sequences (1,326‍ ‍bp) and *nxrB1* gene sequences (302 bp) were sequenced by Fasmac.

### Phylogenetic ana­lysis

A phylogenetic ana­lysis of strain A67 based on the bacterial 16S rRNA gene and *nxrB1* gene was conducted. Evolutionary history was inferred using the maximum likelihood method. The Jukes-Cantor model (Jukes and Cantor, 1969) was used for bacterial 16S rRNA gene sequences and the Kimura 2-parameter model (Kimura, 1980) for *nxrB1* gene sequences. Evolutionary ana­lyses were conducted using MEGA X software (Kumar *et al.*, 2018).

### Data availability

The 16S rRNA gene sequence of *Nitrobacter* sp. A67 was deposited at the DNA Data Bank of Japan (DDBJ) under accession number LC702421. The *nxrB* gene sequence of *Nitrobacter* sp. A67 was deposited at DDBJ under the accession number LC702422.

## Results and Discussion

### A source culture to isolate novel NOB from agricultural soil

The experimental procedure for a community ana­lysis of an isolation source and the isolation of novel NOB is shown in a flow chart (Fig. 1). AOM and NOB in agricultural soil were enumerated using two media containing urea: ULAC medium (pH 5.5) and UL medium (pH 7.6) (Table 1). NO_3_^–^ was detected in all 12 nitrification-positive tubes for a MPN-enumerating nitrifying population using ULAC medium (pH 5.5), indicating AOM+NOB (and/or comammox) positivity. Among 20 nitrification-positive tubes when UL medium (pH 7.6) was used for MPN enumeration, NO_3_^–^ was detected in one out of 20 nitrification-positive tubes, indicating AOM+NOB (and/or comammox) positivity. In the remaining 19 tubes, NO_3_^–^ was absent, whereas NO_2_^–^ was detected, indicating AOM positivity and NOB negativity. Comparisons of the MPN estimates of AOM using two media revealed 61-fold higher estimates (1.7×10^5^ [g wet soil]^–1^) in UL medium (pH 7.6), the pH of which was markedly closer to that measured with the soil sample used (pH 7.2). Regarding the MPN score of NOB, ULAC medium (pH 5.5) recovered 140-fold more (2.8×10^3^ [g wet soil]^–1^) than UL medium (pH 7.6). However, medium pH during cultivation was not monitored. This result suggests that NOB in the soil sample adapted to acidification in their microhabitat. If this is the case and if medium corresponding to the bulk pH of soil was used, ‘acidophilic and/or acid-tolerant’ NOB may have been overlooked. Therefore, NOB isolated from enrichment cultures with conventional methods may not necessarily reflect the ecology of NOB. The NOB isolation strategy used in the present study (Fig. 1) overcame these essential issues in microbial ecology and related ecophysiology. One of the 12 AOM+NOB-positive cultures in ULAC medium was designated as culture A31. The nitrification activity of culture A31 was maintained even after being subcultured for 1 year. Therefore, culture A31 was used as an isolation source for NOB that adapt to an acidic environment.

**Fig. 2.**
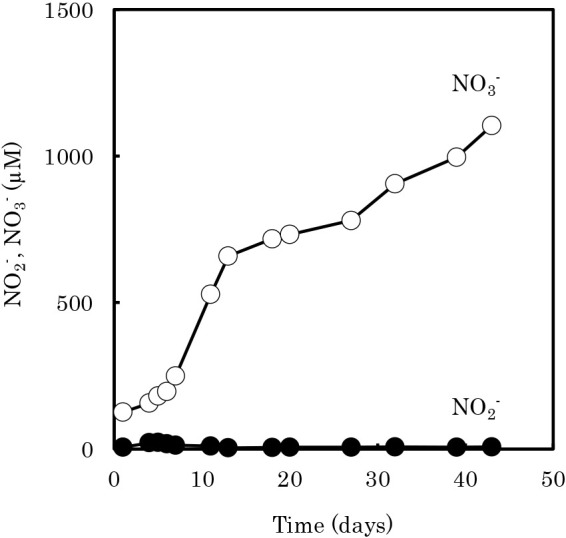
Nitrification activity and microbial community ana­lysis of culture A31 (isolation source of strain A67). NO_2_^–^ (closed circle) and NO_3_^–^ (open circle) in culture A31 by subculturing on ULAC medium (pH 5.5) containing 0.75‍ ‍mM urea. NO_2_^–^ was not detected during the incubation, and complete nitrification from urea to NO_3_^–^ was observed. The experiment was conducted without replication. However, re-experiments provided similar results (data not shown).

The nitrification process from urea to NO_3_^–^ in culture A31 was observed by measuring NO_2_^–^ and NO_3_^–^ concentrations (Fig. 2). The accumulation of NO_2_^–^ was not observed. In community ana­lyses of culture A31 based on the 16S rRNA gene, the ratios of the sequences affiliated with the genus *Nitrososphaera* (AOA) and the genus *Nitrobacter* (NOB) were 20.58 and 0.12%, respectively. Sequences related to comammox *Nitrospira* were not detected.

Some AOA in acidic soils are ureolytic and may generate NH_3_ directly from urea (Lu and Jia, 2013). The adaptive growth of *Nitrososphaera*-like AOA (1.1b group) in acidic soil has also been reported (Wang *et al.*, 2014a). In sequences related to the genus *Nitrososphaera* in culture A31, the closest strain was *Nitrososphaera viennensis* EN76 isolated from garden soil (CP007536). A previous study found an urease gene cluster in the genome of strain EN76 and suggested that this archaeon used urea as a sole energy source instead of ammonia (Tourna *et al.*, 2011). The acidic nitrification process in the A31 culture may be maintained through the oxidation of NH_3_ to NO_2_^–^ by *Nitrososphaera* (AOA) and the oxidation of NO_2_^–^ to NO_3_^–^ by *Nitrobacter* (NOB). Since the concentration of NO_2_^–^ produced was very low (Fig. 2), the concentration of free nitrite may have been below the inhibitory value of NOB based on chemical equilibria at acidic pH (Fig. S1).

Additionally, ammonia as a sole energy source was used to enrich comammox and NOB *Nitrospira* in a previous study (Takahashi *et al.*, 2020). In the present study, urea instead of ammonia was supplied to enrich AOA and NOB. Although the abundance of AOA and comammox *Nitrospira* in the sampling site in the present study remains unknown, a difference in ammonia or urea availability under acidic conditions may contribute to the selective enrichment of AOA and comammox *Nitrospira*.

### Isolation and phylogenetic ana­lysis of NOB

The dilution-extinction procedure was applied to culture A31 for the isolation of NOB. NUAC medium (pH 5.5) containing 100‍ ‍μmol L^–1^ NaNO_2_ was used. After an incubation for 25 days, 2 out of 400 cultures were positive for NO_3_^–^ (*i.e.*, positive for NOB), and an aliquot of one of the two, designated as culture A67, was transferred (10% [v/v]) to 13.5‍ ‍mL of freshly prepared NUAC medium (pH 5.5) containing 100‍ ‍μmol L^–1^ NaNO_2_. After subculturing A67 (10% [v/v]) for 2 years on 90‍ ‍mL NUAC medium (pH 5.5) containing 0.3‍ ‍mmol L^–1^ NaNO_2_ in a 200-mL Erlenmeyer flask, a purity check of culture A67 was conducted by investigating (1) whether the sequences of 16S rRNA gene clones were identical, (2) the consistency of cells stained for FISH, and (3) the growth of heterotrophs. Candidate culture A67 was a pure culture because (1) all 94 clones of the 16S rRNA gene sequences formed a single OTU with a sequence identity of 98.7% or higher, (2) all bacterial cells stained with the EUB338 probe and targeted cells stained with the Nbac-154 probe were consistent (Fig. S2), and (3) there was no growth of heterotrophs. Collectively, these results confirmed that we obtained a pure culture of NOB designated as strain A67.

**Fig. 3.**
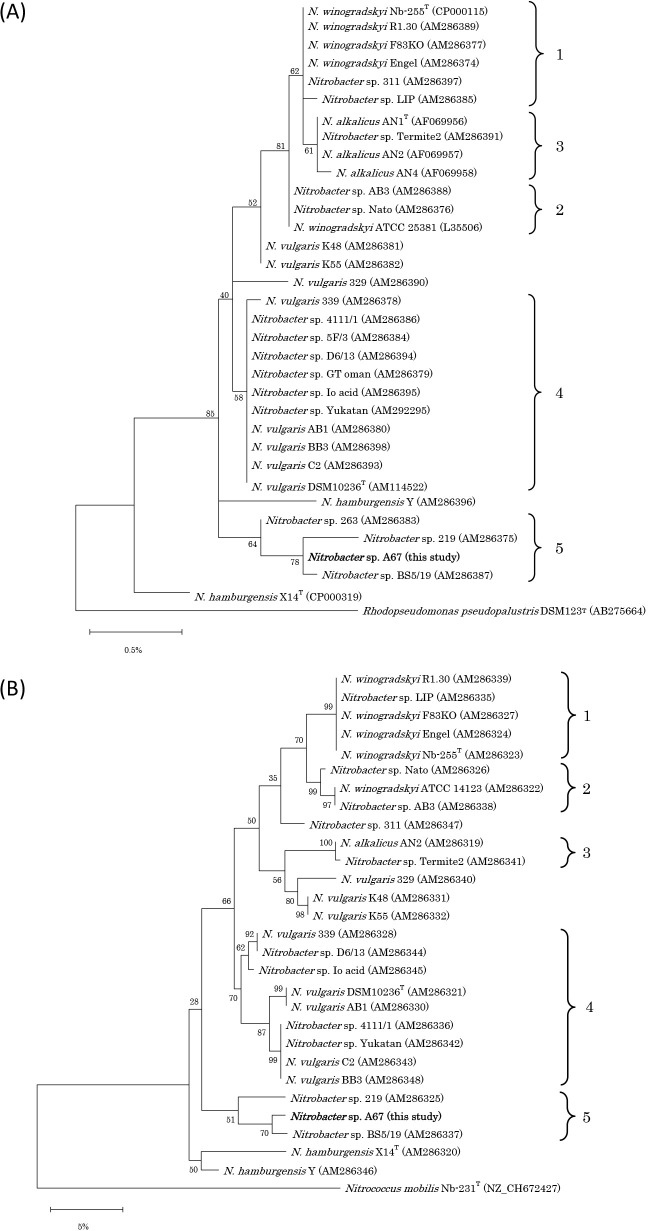
(A) Phylogenetic tree based on 16S rRNA gene sequences of the genus *Nitrobacter*. *Rhodopseudomonas pseudopalustris* DSM123^T^ was inserted as an outgroup. The tree was constructed using the maximum-likelihood method. Bootstrap values at the branch nodes were iterated 1,000 times. The strain obtained in the present study is shown in bold. The scale bar corresponds to 0.5% estimated sequence divergence. Accession numbers are shown to the right of the organism names in brackets. Cluster numbers from 1 to 5 are shown based on a previous study (Vanparys *et al.*, 2007). (B) Phylogenetic tree based on the *nxrB1* gene sequences of the genus *Nitrobacter*. A *Nitrococcus mobilis* Nb-231^T^ *nxrB* homologue was used as an outgroup. The tree was constructed using the maximum-likelihood method. Bootstrap values at the branch nodes were iterated 1,000 times. The strain obtained in the present study is shown in bold. The scale bar corresponds to 5% estimated sequence divergence. Accession numbers are shown to the right of the organism names in brackets. Cluster numbers from 1 to 5 are shown based on a previous study (Vanparys *et al.*, 2007).

The 16S rRNA gene sequence of *Nitrobacter* sp. A67 (1,326 bp) was classified into cluster 5, as defined by Vanparys *et al.* (2007) (Fig. 3A), and sequence identity was‍ ‍99.92% with *Nitrobacter* sp. BS5/19, 99.77% with *Nitrobacter* sp. 263, and 99.70% with *Nitrobacter* sp. 219. Sequence identity with the type strains was 99.39% with *N. winogradskyi* Nb-255, 99.32% with *Nitrobacter alkalicus* AN1, 99.39% with *Nitrobacter vulgaris* DSM10236, and 98.94% with *Nitrobacter hamburgensis* X14.

The *nxrB1* gene sequences of *Nitrobacter* sp. A67 (302‍ ‍bp) were also classified into cluster 5, as defined by Vanparys *et al.* (2007) (Fig. 3B), and sequence identity was 96.10% with *Nitrobacter* sp. BS5/19 and 94.98% with *Nitrobacter* sp. 219. Sequence identity with the type strains was 92.36% with *N. winogradskyi* Nb-255, 93.82% with *N. vulgaris* DSM10236, and 91.44% with *N. hamburgensis* X14.

Vanparys *et al.* (2007) summarized that strains 263, BS5/19, and 219 were distantly related to the strains of all currently known *Nitrobacter* species and, thus, may belong to another species. Since *Nitrobacter* sp. A67 was classified into cluster 5 and the *nxrB1* gene had low sequence identity with the previously isolated strains, *Nitrobacter* sp. A67 may be a novel species; however, genome sequencing in a future study may clarify whether strain A67 is a new species of *Nitrobacter*.

### Physiology of *Nitrobacter* sp. A67

**Fig. 4.**
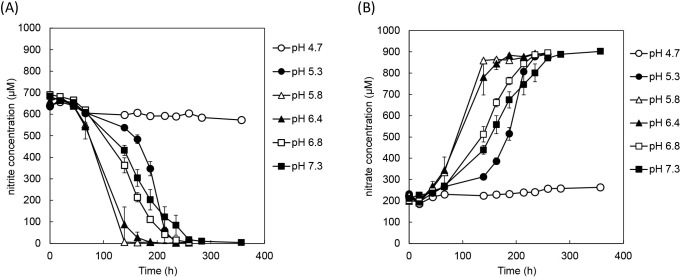
NO_2_^–^ oxidation rate of *Nitrobacter* sp. A67 at various pH. (A) Nitrite consumption, (B) nitrate production. Data show average values obtained in duplicate.

NO_2_^–^ consumption and NO_3_^–^ production by *Nitrobacter* sp. A67 from pH 3.7 to 7.3 was monitored for 17 days. The chemical destruction of NO_2_^–^ at pH 3.7 and 4.2 was observed in negative control (non-inoculated) experiments; however, NO_2_^–^ was stable at other pH. The optimum pH for NO_2_^–^ oxidation by strain A67 was 5.8–6.4, while NO_2_^–^ oxidation at pH 4.7 was not observed (Fig. 4). A previous study reported that the NO_2_^–^ oxidation rate of *Nitrobacter* sp. Io acid was optimum at pH 5.5 and declined with decreases in pH (Hankinson and Schmidt, 1988). This finding suggests that *Nitrobacter* sp. A67 and *Nitrobacter* sp. Io acid are moderately acidophilic NOB. Cluster classification in the genus *Nitrobacter* was based on the 16S rRNA and *nxrB1* genes (Fig. 3). Phylogenetically, *Nitrobacter* sp. Io Acid and strain A67 were classified as clusters 4 and 5, respectively. The acidophilic or acid-tolerant properties of the genus *Nitrobacter* may depend on strains. It currently remains unknown whether *Nitrobacter* strains adapting to acidic conditions concentrate in specific clusters within the phylogeny.

**Fig. 5.**
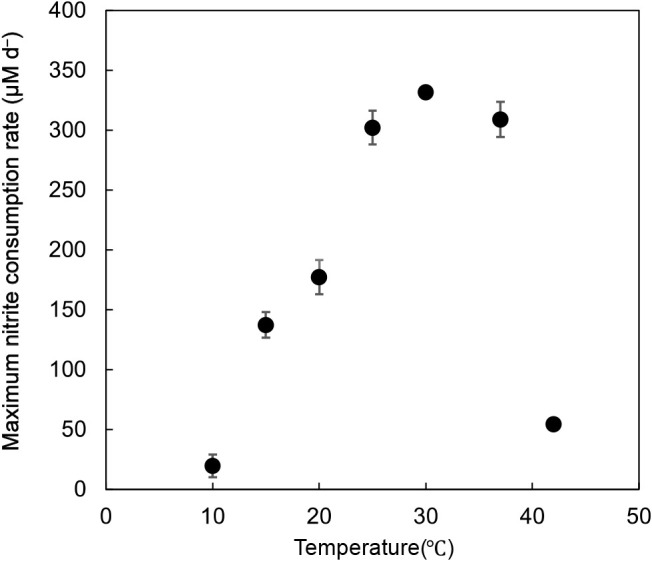
Optimum temperature of *Nitrobacter* sp. A67. The maximum NO_2_^–^ oxidation rate was defined as the average value of the three cultures. Error bars show standard deviations (*n*=3).

Regarding temperature, *Nitrobacter* sp. A67 showed high nitrite consumption rates from 25°C to 37°C. The optimum temperature for nitrite consumption by strain A67 was 30°C, whereas nitrite consumption at 10°C was not observed (Fig. 5). Additionally, *Nitrobacter* sp. A67 did not exhibit urea degradation activity (data not shown). To the best of our knowledge, urea degradation by *Nitrobacter* strains has yet to be reported.

### Ecological aspects of NOB

*Nitrobacter* sp. A67 cells were cultured at pH 6.1 and pH 7.1 and then stained using DAPI. The cells of *Nitrobacter* sp. A67 dispersed at pH 6.1, but formed dense aggregates at pH 7.1 (Fig. S3). *Nitrobacter* strain NHB1 performs nitrification by forming aggregates surrounding AOB at pH 4.0 and protecting AOB from the toxicity of nitrous acid (HNO_2_) (De Boer *et al.*, 1991). *Nitrobacter* sp. A67 may protect itself from external stress and maintains its NO_2_^–^ oxidation activity by forming aggregates at neutral pH. Further studies on *Nitrobacter* sp. A67 are required to elucidate the relationship between pH and aggregation. Additionally, *Nitrobacter* sp. A67, one of the NOB strains that accommodates moderately acidic pH, may provide insights into the mechanisms underlying NO_2_^–^ oxidation activity in low pH environments. Genome information on strain A67 needs to be obtained in future studies in order to elucidate these mechanisms. The genes and transporters necessary for acidic adaptation may be found in the genome of strain A67. Apart from the genus *Nitrobacter*, two strains affiliated with the genus *Nitrotoga*, a group of ecologically important NOB, had an optimum pH in the slightly acidic range, whereas the majority of other cultures of *Nitrobacter* and *Nitrotoga* showed optimal pH in the neutral to slightly alkaline range (Spieck *et al.*, 2021). Therefore, the adaptation of *Nitrobacter* and *Nitrotoga* to low pH may be specific to each strain. Since nitrite oxidation occurs in acidic environments, including soils, the isolation of acid-tolerant or acidophilic NOB is essential for understanding the physiology of these NOB.

## Citation

Endo, Y., Fujitani, H., Kaneko, A., Ninomiya, T., Umezawa, C., Kuroiwa, M., and Suwa, Y. (2024) Isolation of a Moderately Acidophilic *Nitrobacter* from a Nitrifying Community Supplied with Urea. *Microbes Environ ***39**: ME24027.

https://doi.org/10.1264/jsme2.ME24027

## Supplementary Material

Supplementary Material

## Figures and Tables

**Table 1.** MPN enumeration of NO_2_^–^ and NO_3_^–^ production after a 42-day incubation of an agricultural soil sample on ULAC medium (pH 5.5) and UL medium (pH 7.6) amended with 0.75‍ ‍mM urea.

dilution	MPN score on^1)^										
	ULAC medium						UL medium				
10^–2^	+	+	+	+	+		*	*	+	*	*
10^–3^	+^2)^	–	+	+	+		*	*	*	*	*
10^–4^	–	–	+	+	+		*	*	*	*	*
10^–5^	–	–	–	–	–		*	–	*	*	*
10^–6^	–	–	–	–	–		–	–	–	–	*
MPN^3)^ of AOM (ratio)	2.8×10^3^ (1)						1.7×10^5^ (61)				
MPN of NOB (ratio)	2.8×10^3^ (140)						0.02×10^3^ (1)				

1) “+”, NO_3_^–^ was detected (≥10‍ ‍μM); “*” NO_2_^–^ was detected (≥10‍ ‍μM). 2) This culture was selected as the isolation source of NOB and designated as culture A31. 3) MPN (g wet soil)^–1^.
