# Supplementary material for: Isolation of a Moderately Acidophilic Nitrobacter from a Nitrifying Community Supplied with Urea
Source: Microbes Environ. 2024 Sep 13;39(3):ME24027. doi: 10.1264/jsme2.ME24027 (PMC11427310; doi:10.1264/jsme2.ME24027)
Supplement: Supplementary file 1 — Supplementary Material [file 39_24027_s1.pdf]

Supplementary material

Materials and Methods

Media

In this study, we used a medium modified from the previously described basal medium C (Suwa *et al*. 1994), by adding 220 mg L^{-1} $NaHCO_3$ as a carbon source in light of the reduced CO_2 supply under acidic conditions and lowering the amount of trace element mixture in ATCC medium #1573 from 1.0 to 0.6 mL L^{-1}. Consequently, modified basal medium C consisted of KH_2PO_4, 725 mg L^{-1}; Na_2HPO_4, 1136 mg L^{-1}; $NaHCO_3$, 220 mg L^{-1}; $MgSO_4{\cdot}7H_2O$, 50 mg L^{-1}; $CaCl_2{\cdot}2H_2O$, 20 mg L^{-1}; Fe-EDDHA, 1 mg L^{-1}; trace element mixture of ATCC medium #1573, 0.6 mL L^{-1}. The ATCC trace element mixture consisted of $Na_2MoO_4{\cdot}2H_2O$ (10 mg), $MnCl_2{\cdot}4H_2O$ (20 mg), $ZnSO_4{\cdot}7H_2O$ (10 mg), $CoCl_2{\cdot}6H_2O$ (0.2 mg), $CuSO_4{\cdot}5H_2O$ (2 mg) in 100 mL of distilled water. To adjust the pH of the medium used in this study, 2 N H_2SO_4 or 1 N NaOH was used.

ULAC medium (pH 5.5), containing 0.75 mmol L^{-1} urea in modified basal medium C, was used for MPN enumeration and subculture of culture A31. To prepare the ULAC medium, two component solutions, A and B, were prepared: KH_2PO_4, Na_2HPO_4, $NaHCO_3$ and urea in solution A, and $MgSO_4{\cdot}7H_2O$, $CaCl_2{\cdot}2H_2O$, Fe-EDDHA, and trace element mixture of ATCC medium #1573 in solution B. The pH was adjusted to 5.5 with 1M HCl when preparing solution A. Each component solution was autoclaved separately, cooled to room temperature, and finally mixed aseptically in a laminar flow hood. Note that urea could be hydrolyzed during autoclave treatment and may produce ammonia (Jiang and Bakken 1999). UL medium (pH 7.6) was also used for MPN enumeration, in which basal medium

(modified basal medium C; pH 7.6) was not amended with 220 mg L^{-1} $NaHCO_3$, but a 0.47 M Na_2CO_3 solution was used for adjusting the pH.

NUAC medium (pH 5.5) containing 100 μmol L^{-1} $NaNO_2$ in modified basal medium C as the sole source of nitrogen and energy for NOB, was used to isolate NOB (pH 5.5). During subculture of *Nitrobacter* sp. A67, $NaNO_2$ was changed to 0.3 or 0.75 mmol L^{-1}. The method for preparing the NUAC medium was the same as that for preparing the ULAC medium.

Purification check

Strain A67 was proven to be pure by (1) examination of 94 16S rRNA gene clones and (2) microscopic observation by fluorescence *in situ* hybridization (FISH), and (3) heterotrophic medium.

(1) The bacterial 16S rRNA gene sequences were amplified by PCR with the primer set 27f/1492r (Table S1) and TaKaRa Ex Taq (TaKaRa Bio, Japan) with the following thermal protocol:94°C for 30 s; 35 cycles of 98°C for 10 s, 56°C for 30 s, and 72°C for 60 s; and 72°C for 5 min. The PCR product was directly ligated into the pCR™4-TOPO® TA vector (Invitrogen, USA) and transformed into One Shot™ TOP10 chemically competent *E. coli*. The clone insert 16S rRNA genes were sequenced by Fasmac (Kanagawa, Japan) and clustered into operational taxonomic units (OTU), with 98.7% sequence identity.

(2) The cell suspension of A67 was fixed in 4% freshly prepared paraformaldehyde solution for 2 h at 4 °C and washed twice with 1×phosphate-buffered saline (PBS). Each sample was stored at -20 °C with ethanol and 1×PBS added in a 1:1 ratio until observation. Ten microliters of the sample was dropped onto a glass slide and air dried at 46 °C for 10 min then stained using fluorescence *in situ* hybridization (FISH), as described in a previous study (Amann *et al*. 1990). The cell suspension of culture A67 was dehydrated in 50%, 80%, and 98% ethanol (3 min each) and the cells were hybridized at 46°C for 3 h with probes. The oligonucleotide probe Nbac-154 (5'-ctaataccggataagccc-3') designed in this study is situated at position 154-171 of the 16S rRNA gene sequence of *Nitrobacter winogradskyi* Nb-255^{T} (NR074324), labeled with hydrophilic sulfoindocyanine dye (Cy3). Furthermore, most bacteria were stained with EUB338 labeled with fluorescein isothiocyanate (FITC) (Table S2). After hybridization, the slide glass was washed for 18 min at 48℃ in wash solution (0.2 M NaCl, 0.1% sodium dodecyl sulfate (SDS), 20 mM Tris/HCl (pH 7.2)), and SlowFade^{TM} Gold antifade reagent (Thermo Fisher Scientific, Waltham, MA). The stained samples were observed under an IX83 inverted microscope (Olympus, Japan).

(3) A67 was inoculated (10% (v/v)) into 1/4-diluted TSB (TSB/4), 1/2-diluted FTG (FTG/2), 1/100-diluted NB (NB/100), and 1/100-diluted R2A (R2A/100) media (Difco, USA) to examine the contamination of heterotrophs. Inoculated TSB/4, FTG/2, NB/100, and R2A/100 were incubated at 25 °C in the dark for 19 days.

Microscopic observation

Nitrobacter sp. A67 was precultured with stirring in 450 mL NUAC medium (pH 6.1 and 7.1) containing 12 mmol L^{-1} $NaHCO_3$ and 1.5 mmol L^{-1} $NaNO_2$ in 1L Erlenmeyer flask at 25 °C at 10% (v/v) for 33 days. After cells in cultures were collected, a cell suspension of *Nitrobacter* sp. A67 was fixed and stored using the method described above. Ten microliters of sample on a glass slide were air-dried at 46°C for 10 min and stained with 10 μL of 1 mg μL^{-1} stock solution of 4',6-diamidino-2-phenylindole, dihydrochloride, solution (DAPI solution) (Dojindo, Japan) at room temperature for 10 min. The slide glass was washed with distilled water and the SlowFade™ Gold antifade reagent (Thermo Fisher Scientific, Waltham, MA). The stained samples were observed under an IX83 inverted microscope (Olympus, Japan).

Table S1. Soil chemical properties

Water contents (%)	pH (H_2O)	TOC (mg L^{-1})	IC (mg L^{-1})	NH_4^+ (mg L^{-1})	NO_2^- (mg L^{-1})	NO_3^- (mg L^{-1})
35.0 ± 1.0	7.22	1116	15.7	0.9	n.d.*	n.d.*

*n.d. = not detectable

Table S2. Oligonucleotide primers for PCR used in this study.

Target	Primer	Sequence（5’-3’）	Reference
All bacterial 16S rRNA gene	27f	AGA GTT TGA TCM TGG CTC AG	Lane 1991
	530f	GTG CCA GCM GCC GCG G	
	1100r	GGG TTG CGC TCG TTG	
	1492r	TAC GGY TAC CTT GTT ACG ACT T	
Nitrobacter-like *nxrB1* gene	nxrB 1F	ACG TGG AGA CCA AGC CGG G	Vanparys *et al.* 2007
	nxrB 1R	CCG TGC TGT TGA YCT CGT TGA	

Table S3. Oligonucleotide probes used for FISH in this study.

Target	Probe	Sequence（5’-3’）	Formamide concentration [%]	Reference
Most bacteria	EUB338	GCT GCC TCC CGT AGG AGT	0-50	Alm *et al*. 1996
Nitrobacter	Nbac-154	CTA ATA CCG GAT AAG CCC	20	This study

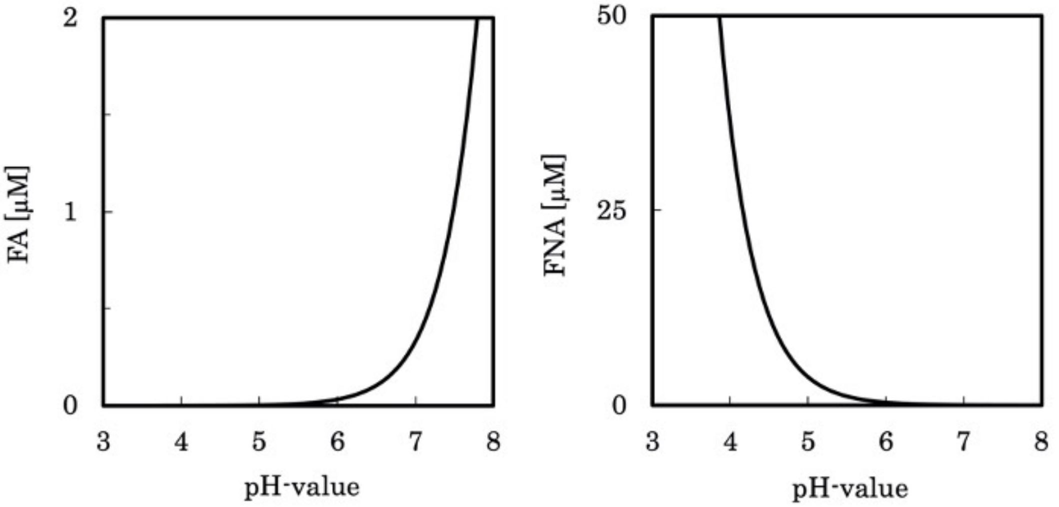

Fig. S1

Free Ammonia (FA) and Free Nitrous Acid (FNA) concentrations under different pH-values based on the equation of Anthonisen *et al.* (1976). FA was calculated as 1 mM of total ammonia concentration (ammonium + ammonia). FNA was calculated as 0.05 mM of total nitrite concentration (nitrite + nitrous acid).

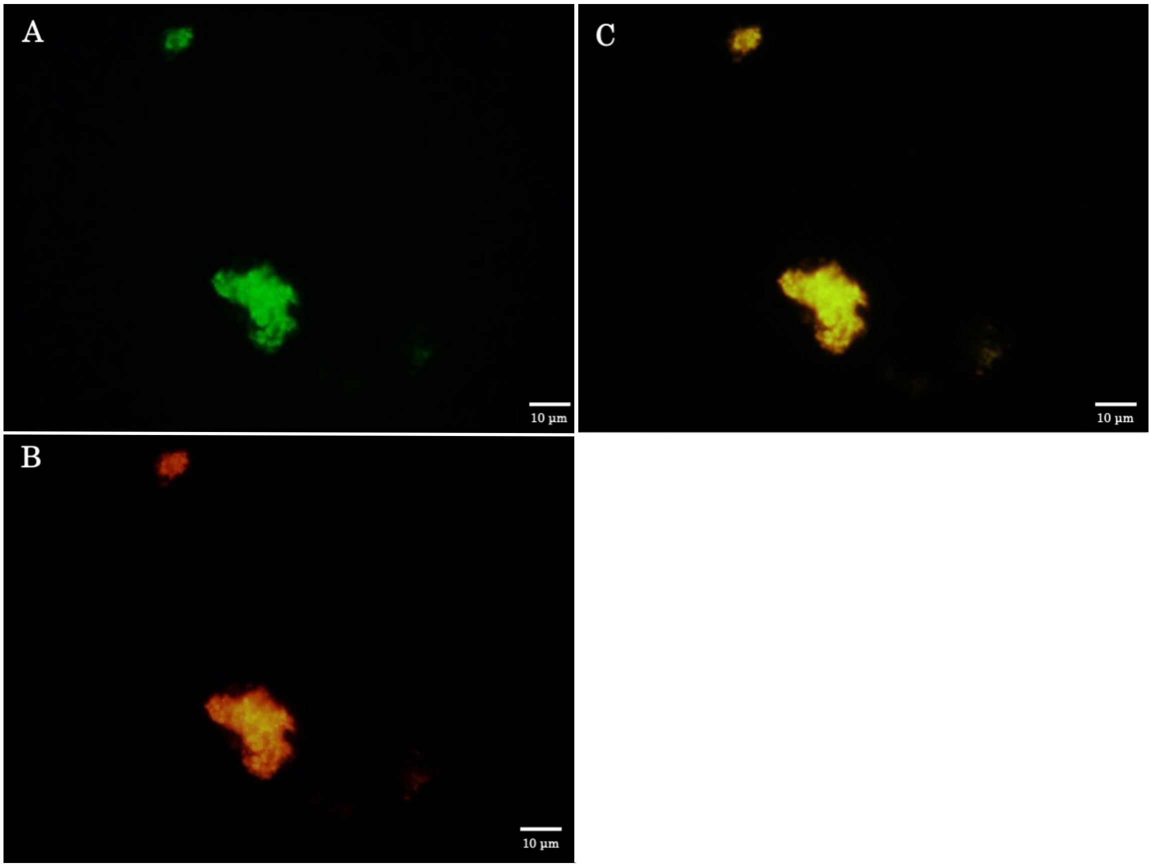

Fig. S2

Fluorescence *in situ* hybridization (FISH) images of culture A67. (A) Most bacterial cells were stained with EUB338 probe labeled with FITC (green), and (B) targeted cells were stained with Nbac-154 probe labeled with Cy3 (red). (C) Two colors were merged into yellow. Scale bar shows 10 µm

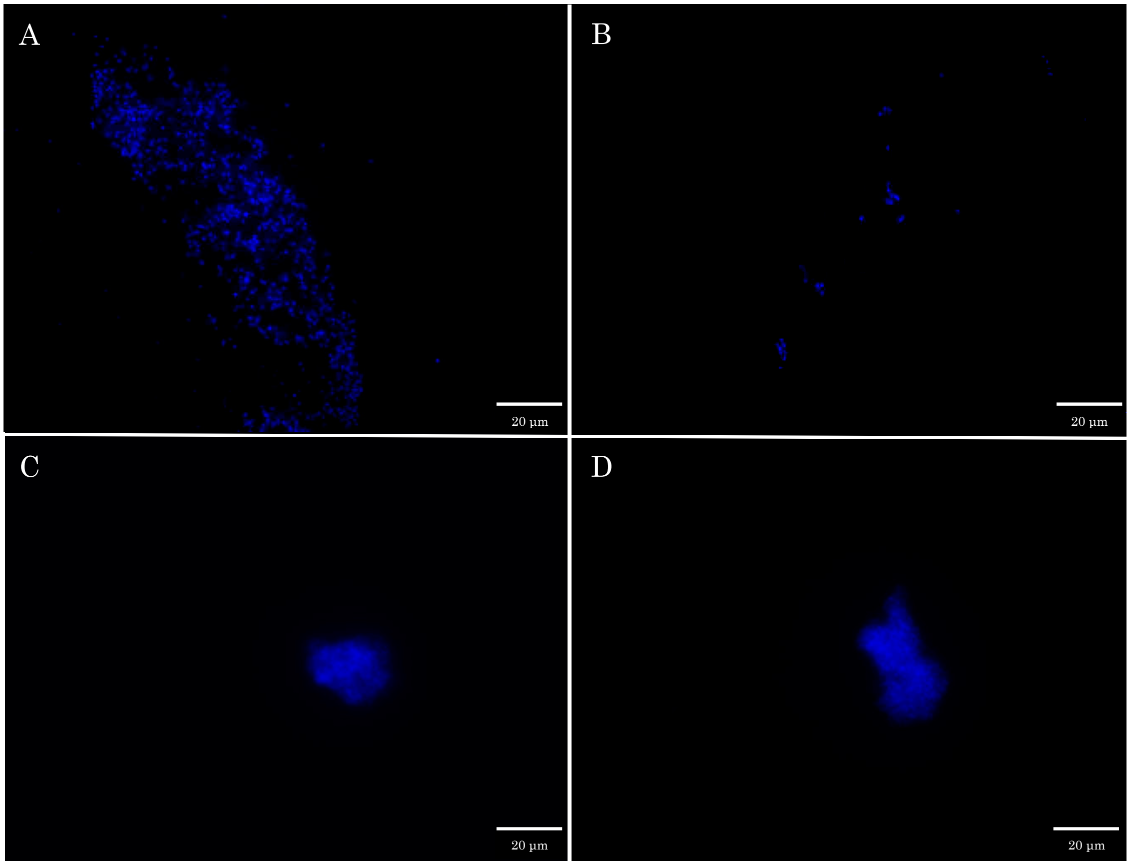

Fig. S3

Microscopic observation of *Nitrobacter* sp. A67 by DAPI (pH 6.1: A, B, pH 7.1: C, D). At pH 6.1, where NO_2^- oxidation activity is high, the cells are dispersed (A, B), but at pH 7.1, where the NO_2^- oxidation activity is considerably low, the cells densely formed aggregates (C, D). Scale bar shows 20 µm

References

Alm, E., Oerther, D., Larsen, N., Stahl, D., and Raskin, L. (1996) The oligonucleotide probe database. *Appl Environ Microbiol* **62**: 3557-3559.

Amann, R., Krumholz, L., and Stahl, D. (1990) Fluorescent-oligonucleotide probing of whole cells for determinative, phylogenetic, and environmental studies in microbiology. *J Bacteriol* **172**: 762-770.

Anthonisen, A.C., Loehr, R.C., Prakasam, T.B.S., and Srinath, E.G. (1976) Inhibition of nitrification by ammonia and nitrous acid. *J Water Pollut Control Fed* **48**: 835-852.

Lane, D.J. (1991) 16S/23S rRNA sequencing. In: Stackebrandt E, Goodfellow M (eds). *Nucleic Acid Techniques in Bacterial Systematics*. Chichester, United Kingdom: Wiley, 115–75.

Jiang, Q.Q., and Bakken, L.R. (1999) Comparison of *Nitrosospira* strains isolated from terrestrial environments. *FEMS Microbiol Ecol* **30**: 171-186.

Suwa, Y., Imamura, Y., Suzuki, T., Tashiro, T., and Urushigawa, Y. (1994) Ammonia-oxidizing bacteria with different sensitivities to $(NH_4)_2SO_4$ in activated sludges. *Water Res* **28**: 1523-1532.

Vanparys, B., Spieck, E., Heylen, K., Wittebolle, L., Geets, J., Boon, N., and De Vos, P. (2007) The phylogeny of the genus *Nitrobacter* based on comparative rep-PCR, 16S rRNA and nitrite oxidoreductase gene sequence analysis. *Syst Appl Microbiol* **30**: 297-308.
